# Study on the Aging Mechanism and Microstructure Analysis of Rice-Husk-Ash- and Crumb-Rubber-Powder-Modified Asphalt

**DOI:** 10.3390/polym14101969

**Published:** 2022-05-12

**Authors:** Yiming Li, Alaaeldin A. A. Abdelmagid, Yanjun Qiu, Enhui Yang, Yanjun Chen

**Affiliations:** 1 Department of Civil Engineering, Northeast Forestry University, Harbin 150040, China; liyiming@nefu.edu.cn; 2 Longjian Road and Brigde Co., Ltd., Harbin 150009, China; longjianyanfa@163.com; 3 School of Civil Engineering, Southwest Jiaotong University, Chengdu 610031, China; yjqiu@home.swjtu.edu.cn (Y.Q.); ehyang@swjtu.edu.cn (E.Y.); 4 Highway Engineering Key Laboratory of Sichuan Province, Southwest Jiaotong University, Chengdu 610031, China

**Keywords:** asphalt binder, rice husk ash, crumb rubber powder, functional group, aging resistance, scanning electron microscope

## Abstract

In this paper, the rice husk ash and crumb rubber powder were used as a combined modifier for asphalt. The impact of the aging on the physical and rheological properties of crumb rubber powder, rice husk ash, and the combined modified asphalt was studied through the rolling thin film oven (RTFO) simulations. A Fourier-transform infrared Spectroscopy (FTIR) test was used to study the aging mechanisms of the combined crumb-rubber-powder- and rice-husk-ash-modified asphalt before and after aging through the changes in functional groups. Impacts of the combined, crumb rubber powder, and rice husk ash modifiers on the anti-aging characteristic of the asphalt binder were analyzed through different aging indices and the variations in intensity of the absorption peaks. According to the combined results, the addition of the combined crumb rubber powder, and rice husk ash could enhance the thermal oxidative aging resistance binder. Moreover, the optimal content of composite modified asphalt was (7% rice husk ash + 10% crumb rubber powder). In addition, the combined modified asphalt binder had all the peaks of neat asphalt, rice-husk-ash-modified asphalt, and crumb-rubber-powder-modified asphalt and no appearance of new peaks. A scanning electron microscope (SEM) test was carried out to observe the microstructure of the combined crumb-rubber-powder- and rice-husk-ash-modified asphalt binders. The obtained result demonstrated that different SEM images showed that the combined crumb rubber powder, and rice husk ash modifiers were uniformly dispersed inside the asphalt binder and consequently leading to format a homogeneous blended binder.

## 1. Introduction

For several decades, with the development of economy and infrastructure construction, the amount of agricultural and industrial waste is increased. The improper treatment and use will lead to environmental pollution. At the same time, the performance and economic cost of building materials are the main concerns of experts and specialists. Therefore, the development and utilization of waste materials to be used as a new kind of building materials has become the consensus of researchers, and it is also the trend of social development. Rice husk and rubber are two kinds of waste materials. Improper disposal of them will not only lead to environmental pollution, but also lead to a waste of resources.

The use of rice husk ash (R) as modifier is relatively new. In our previous studies, it was stated that the use of R and crumb rubber powder (P) has great positive effects on the high-temperature performance of the asphalt binder [1,2]. Cai et al. [3] investigated the influence of R as representative of the biomass ashes on the basic asphalt binder performance, and they concluded that the basic performance has been improved. Xue et al. [4] used two types of biomass ashes, wood sawdust ash (WSA) and R as modifiers. They reported that both WSA- and R-modified asphalt binders exhibited positive effects on physical properties and rutting performance. In addition, considerable efforts have been made to use and utilize the R for the asphalt mixture. A study has been conducted by M. Arabani and S.A. Tahami [5], their study focused on the impacts of adding R to the HMA mixture. They detected that adding R had improved fatigue performance and rutting strengths of HMA concrete. Lu et al. [6] investigated the asphalt mixture containing polystyrene-butadiene-styrene (SBS) and R. They concluded that the addition of SBS and R had improved the high temperature performance of the mixture. Ameli et al. [7] studied the characteristics of asphalt mixture after replacing the hydrated lime filler with R. They stated that using R could enhance the asphalt mixture Marshall stability.

From the 1930s, the utilize of rubbers from waste rubbers as an additive in the asphalt mixture [8,9,10,11,12,13,14,15]. Because of the ability of crumb rubber powder (P) to enhance the asphalt binder and mixture performance [16,17,18,19,20,21,22,23,24,25], therefore, using of crumb rubbers recently increased widely in construction pavement as a probable solution in minimizing waste tires and the disposal areas. Many researchers used composite-modified asphalt containing P and different modifiers, and they examined the characteristics of the composite-modified asphalt through number of micro-analysis methods and property tests [25,26,27,28,29,30,31,32,33].

Aging is a phenomenon that influence the asphalt binder in terms of physical, rheological, and chemical properties. Aging can be taken place in various stages during construction, transportation, and production of asphalt pavement (short- and long-term aging). Aging on short term takes place because of loss of volatile components and oxidation during the asphalt mixture paving and production when asphalt exposed to air and temperature. While the continuous of oxidation leads to the long-term aging. Many studies used Fourier-transform infrared spectroscopy (FTIR) to assess the impact of aging on the chemical properties, whereas the physical and rheological properties were evaluated through aging indices [34,35].

However, all the above-mentioned studies concerned and dealt with the evaluation of modified asphalt binder performance, and there were quite limit studies evaluated the anti-aging, aging mechanism, and microstructure analysis of asphalt binder modified with R, and the combined modifier of rice husk ash and crumb rubber powder (R/P). Moreover, the application of using R in the field of pavement construction is required because the huge amount of rice husk that produced every year by each country. Therefore, this research work aims to promote a good understanding the anti-aging properties of asphalt containing R/P, R, and P at high temperatures via aging indices. Furthermore, Fourier-transform infrared spectroscopy (FTIR) and scanning electron microscope (SEM) tests were utilized to investigate the effects of R/P, R, and P on the aging mechanism of the asphalt binder and to analyze the microstructure of R/P-, R-, and P-modified asphalt binders.

## 2. Materials and Methods

### 2.1. Materials

#### 2.1.1. Asphalt Binder

The asphalt type is that selected to prepare the modified asphalt binder in the study is AH-70 paving asphalt according to Chinese classification, which is equivalent to 60/80 penetration graded asphalt binder, and it was purchased from Shanghai. The physical characteristics of the neat asphalt are tabulated in Table 1.

#### 2.1.2. Preparation of Rice Husk Ash (R)

**Figure 1:**
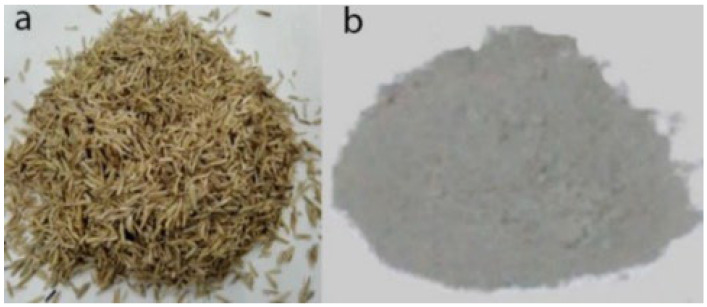
(**a**) Rice husk, and (**b**) rice husk ash.

Rice husk in this work was brought from Harbin city, China. In order to prepare the R, rice husk was burned for two hours at temperature of 650 °C to avoid the carbonization [36]. Then, to form a fine powder, grinding ball mill was used for 30 min. Finally, the 0.075 mm sieve size was used to obtain the required R. Figure 1 displays rice husk and obtained R, and its physical properties and chemical constitutions are shown in Table 2.

#### 2.1.3. Crumb Rubber Powder (P)

**Figure 2:**
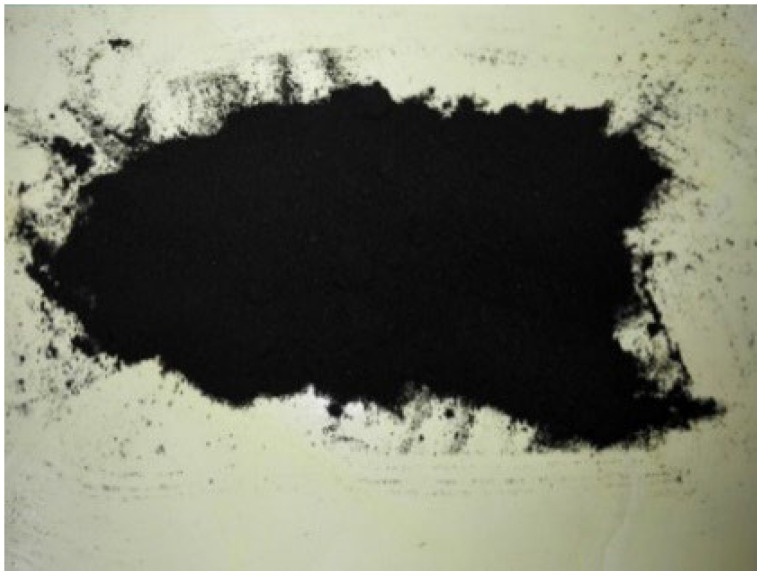
Crumb rubber powder.

**Figure 3:**
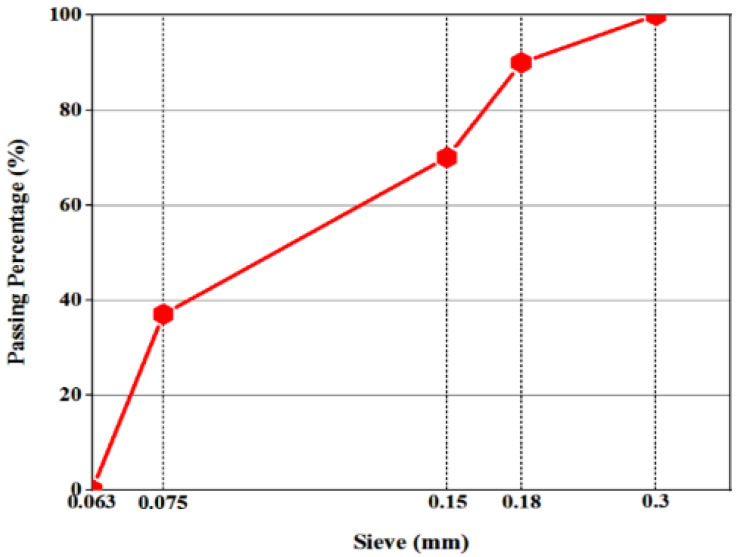
Crumb rubber gradation.

P materials in this work (Figure 2) were purchased from Beijing. Table 3 illustrates the physical properties and chemical constitutions. Figure 3 exhibits crumb rubber gradation.

### 2.2. Sample Preparation

#### 2.2.1. Preparation of Modified Asphalt

To prepare asphalt binders modified with R, P, and R/P, three concentrations of 1%, 4%, and 7% of R were added, and 5%, 10%, and 15% of P by weight of the asphalt binder. Firstly, 500 g of neat asphalt was heated till it reached the molten state; thereafter, various concentrations of R and P were added slowly to the neat asphalt using a mechanical mixer for 15 min at 1000 rpm as the speed. Afterwards, samples were stirred for 20 min at temperatures of 155 ± 5 °C with a speed of 3000 rpm for R [5] for 45 min at a temperature of 177 ± 5 °C with a speed of 700 rpm for P [37,38] using the high-speed mixer. For R/P samples, 5% P was added to the neat asphalt, subsequently, through using a mechanical mixer for 15 min at 1000 rpm as the speed. Afterwards, samples were stirred for 30 min at temperatures of 177 ± 5 °C with a speed of 700 rpm through using the high-speed mixer. Afterward, 1% R was added, and samples were stirred at a temperature of 155 ± 5 °C for 30 min with a speed of 3000 rpm using the high-speed mixer. In order to exclude the air from mixtures, the mechanical mixer was used for 10 min at low speed. A similar procedure was used for (5% P + 4% R), (5% P + 7% R), (10% P + 1% R), (10% P + 4% R), (10% P + 7% R), (15% P + 1% R), (15% P + 4% R), and (15% P + 7% R).

#### 2.2.2. Preparation of Aged Binder

To scrutinize the aging behavior, the rolling thin film oven (RTFO) test was implemented based on ASTM D 2872 standard on the neat asphalt and R/P-, R-, and P-modified asphalt.

### 2.3. Test Methods

#### 2.3.1. Conventional Physical Tests

A penetration test, softening point test, and rotational viscosity test were carried out based on ASTM D5, ASTM D36, and ASTM D4402, respectively.

The goal of utilizing the penetration test was to analyze the hardness of asphalt binder, using a standard loaded needle with a mass of 100 g to measure the vertical depth value at a temperature of 25 °C.

The softening point is a test that determines the temperature at which an asphalt binder achieves a certain level of viscosity. In the test, a steel ball is put on a binder disc that gradually raises the temperature at a rate of 5 ± 0.5 °C per minute till it becomes soft enough for the ball to fall 2.5 cm

At high temperatures, a rotational viscometer (RV) is often used to determine the viscosity of asphalt binder. It is one of the most important characteristics since it indicates the binder’s capacity to be pumped and handled in a hot mixing facility. The viscosity readings were obtained using a Brookfield rotating viscometer and 10.5 g of asphalt binders. The viscosity values of the samples were measured using a spindle No. 27 at a temperature of 135 °C in our investigation.

#### 2.3.2. Storage Stability

The storage stability was used to detect whether phase separation will develop in modified asphalt. The sample was placed into a toothpaste tube made of aluminum (140 mm in height and 25 mm in diameter). The tube was sealed and placed vertically in a 163 °C oven for 48 h. The different between the softening points of the top and bottom parts were measured as the storage stability

#### 2.3.3. Dynamic Shear Rheometer (DSR)

The rheological characteristics of asphalt binder are generally evaluated through the DSR test. In this work, the DSR test was made under controlled strain at temperatures changing from 52 °C to 82 °C with the frequency of 10 rad/s (1.59 Hz). The diameter plate and the gap were 25 mm and 1 mm, respectively [SHRP-A-365].

#### 2.3.4. Aging Indices

Aging indices are used to assess the aging performances of asphalt binder. The proportion of properties of asphalt binder after the aging occurred to the same properties before aging is known as aging index [39]. Indices of aging were determined from physical and rheological characteristics. Indices of aging can be measured via Equations (1)–(4) [35,40,41,42,43,44].

(1) SI=Aged Softening point−Unaged Softening point

SI represents softening point increment.

(2) $$\mathrm{PI}=\frac{\mathrm{Aged}\;\mathrm{penetration}\;\mathrm{value}}{\mathrm{Unaged}\;\mathrm{penetration}\;\mathrm{value}}$$

PI represents penetration aging ratio.

(3) $$\mathrm{VI}=\frac{\mathrm{Aged}\;\mathrm{viscosity}\;\mathrm{value}-\;\mathrm{Unaged}\;\mathrm{viscosity}\;\mathrm{value}}{\mathrm{Unaged}\;\mathrm{viscosity}\;\mathrm{value}}$$

VI represents viscosity aging index.

(4) $$\mathrm{AIR}\;=\frac{\mathrm{Aged}\;\mathrm{rutting}\;\mathrm{factor}\;\left(G*/\sin\right)}{\mathrm{Unaged}\;\mathrm{rutting}\;\mathrm{factor}\;\left(G*/\sin\right)}$$

AIR represents aging index of (G∗/sinδ).

#### 2.3.5. Fourier-Transform Infrared Spectroscopy (FTIR)

For inorganic or organic materials, Infrared absorption spectroscopy can be considered as important tool or method to detect and identify the functional group (chemical bond). Thus, the FTIR analysis is widely utilized to identify the organic chemical existing in the matrices. With the range from 4000 to 500 cm^−1^, FTIR (Nicolet is 50) was used to conduct this test in the work. The binders were melted in carbon disulfide with concentration of 5 wt.%, thereafter they were dropped in the potassium bromide window pan and dried before samples were tested.

#### 2.3.6. Scanning Electron Microscope (SEM)

**Figure 4:**
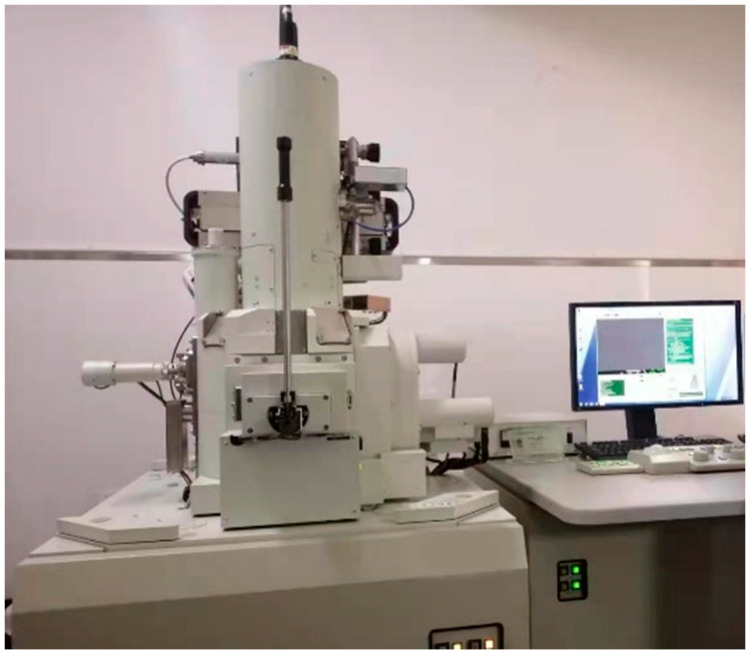
SEM device.

SEM is a multiple characterization tool that allows to observe and analyze the inorganic or organic material on a micro to the nano meter scale. SEM was utilized to scrutinize the micromorphology of all binders. The micron-scale morphology of all samples was visualized through using a JSM-7500F scanning electron microscope (SEM). A thin coating of metal was sprayed on the sample under vacuum conditions after it was fixed on conductive adhesive tape. The specimen with observation was then placed in the sample chamber, and the topography of the sample was observed through the scanning electron microscope. Figure 4 displays the SEM machine used in the work.

## 3. Results and Discussion

### 3.1. Physical Properties

**Figure 5:**
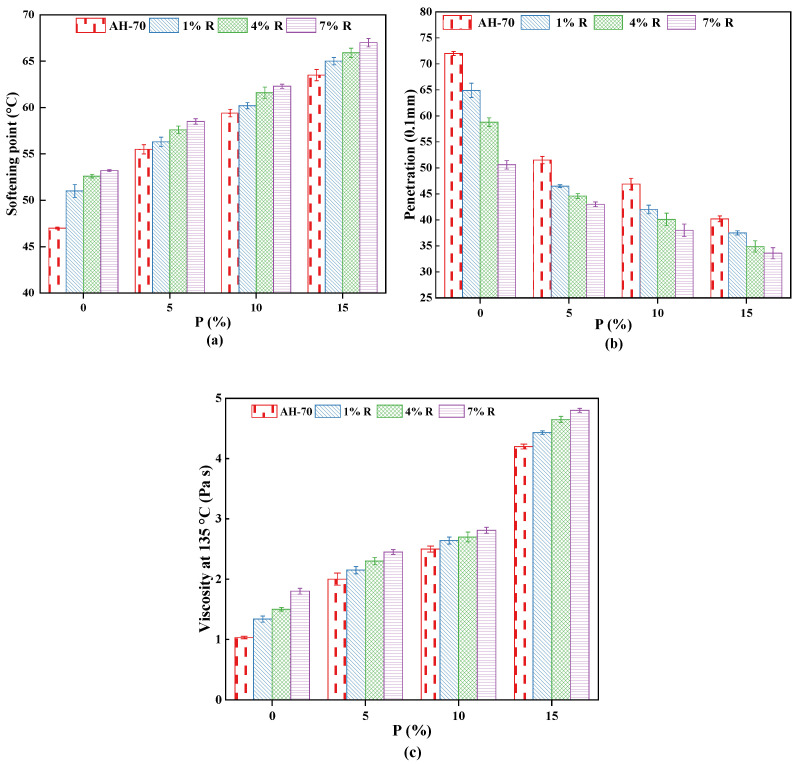
Effect of R/P, R, and P content on (**a**) softening point, (**b**) penetration, and (**c**) viscosity.

The change of softening point, penetration, and viscosity of neat asphalt and R/P-, R-, and P-modified asphalt binder with various dosages of R (1%, 4%, 7%) and P (5%, 10%, 15%) are shown in Figure 5. It can be seen from Figure 5 that with the increment of R/P, R, and P content, the softening point and viscosity of modified asphalt binders increased rapidly, which was positively correlated with R/P, R, or P content, whereas penetration correspondingly decreases. This implies that the use of R and P could improve the thermal stability of neat asphalt and the impact of enhancement is clearly at high dosages of R and P content. Moreover, it is important to mention that values of viscosity rapidly increased especially with high concentration. The reason for this phenomenon is attributed to the R particles affect during blending leading to an increase in the viscosity value. Moreover, the absorption of the binder’s aromatic oil lead swelling of the P particles. The swollen P particle raised the hardness to flow since swollen P particles compared with those that were not swollen occupied more space.

### 3.2. Storage Stability

The asphalt binder modified with high concentration of R/P failed to meet the value 2.2. Thus, care should be used while storing the R/P-modified binder at high temperatures for long period of time. It would be preferred to utilize the R/P-modified binder to prepare the asphalt mixture as soon as it is made, but if this is not possible and it must be kept, the R/P-modified binder must be stored with agitation to ensure material homogeneity.

### 3.3. Rutting Parameter G*/sin (δ)

**Figure 6:**
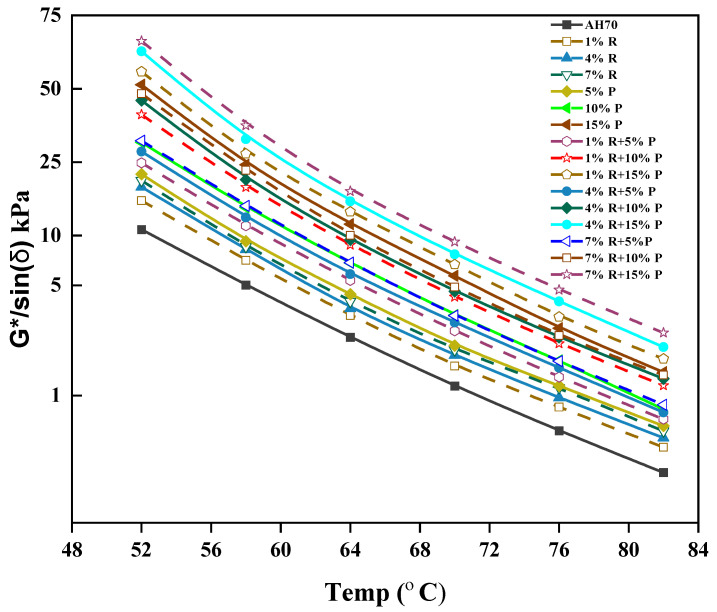
Effect of R/P, R, and P Content on Rutting Parameter G*/sin (δ).

At high temperature, G*/sin (δ) is used for evaluating the resistance of asphalt binder against permanent deformation. Figure 6 displays the values of G*/sin (δ) for various kinds of modified asphalt binder with the changes in test temperature. From Figure 6, the G*/sin (δ) of each group of samples had a decreasing trend when the temperature was raised. This was by reason of the higher temperature caused the asphalt binder to soften, and the viscous portion to raise. It can be observed from Figure 6 that the decreasing trend gradually slowed down, suggesting that the asphalt binder performance at high temperature gradually declined and the influence on it gradually decreased when the temperature rose to a certain high-temperature state. The difference in high temperature performance could be noticed by comparing the G*/sin (δ) of various types of modified asphalt binders. A higher G*/sin (δ) of asphalt suggested a better ability to resist deformation. Moreover, the addition of R/P, R, and P increased the G*/sin (δ). The more R/P, R, and P content, the higher the G*/sin (δ). Asphalt binder with high G*/sin (δ) value displays better rutting resistance due to reduce the dissipated energy amount after any loading cycle. This premise is depended on the supposition that rutting is a stress-controlled, cyclic loading phenomenon. The values of G*/sin (δ) climbed with adding of R/P, R, and P concentrations, which demonstrated that the addition of R/P, R, and P to neat asphalt stiffened the asphalt binder and then enhanced the asphalt binder resistance against permanent deformation. This is because R and P have high surface area reinforcing the binder and raise the cohesive between binders. This phenomenon causes a raise in the adhesion and performance characteristics and therefore enhanced the asphalt resistance against rutting. From Figure 6, the G*/sin (δ) value of R/P-modified asphalt with high content (7% + 15%) was higher than that of other asphalt samples at various temperatures. The G*/sin (δ) of P-modified asphalt binder also had high values at different temperatures. With the rise of temperature, the G*/sin (δ) value of 15% P-modified asphalt binder was gradually close to the G*/sin (δ) value of (1% + 15%) R/P-modified asphalt binder, which displays that the adding of P modifier improved the high temperature rheological properties of the binder to a certain extent, and the high temperature properties of R/P-modified asphalt binders were also significantly enhanced. However, the G*/sin (δ) values of the R-modified asphalt binder were slightly higher than those of neat asphalt at the same temperature, but lower than that of R/P- and P-modified asphalt. Not surprisingly, asphalt binder modified with R/P and P demonstrated the best high-temperature deformation resistance, and these results in line with many studies reported that the addition of P to the asphalt binder has a significant impact on the binder’s high temperature performance as indicated by its upper performance grade (PG) temperature. This is due to P, which can fully expand in asphalt and form a cross-linking structure to improve the connection between asphalt components.

The study showed that using R/P, R, and P positively impact the resistance rutting of asphalt binders at high temperatures.

### 3.4. Aging Indices Evaluation

#### 3.4.1. Short-Term Aging Effect on the Softening Point

**Figure 7:**
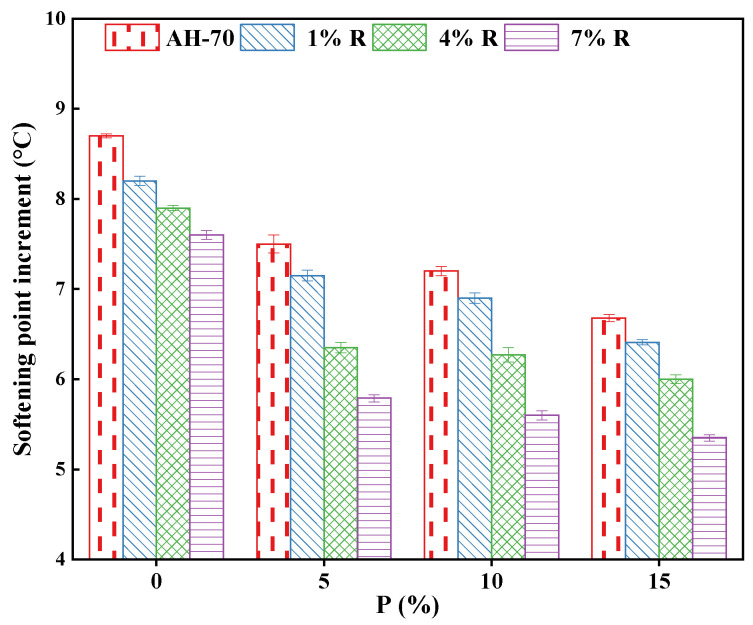
Softening points increment.

The SI of asphalt reflects the rise in the softening point before and after aging, which is expressed by the difference between the softening point after aging and the softening point before aging. The smaller the SI is, the better the aging resistance of asphalt is. Figure 7 illustrates the SI of neat asphalt and R/P-, R-, and P-modified samples. Moreover, the SI values of the neat asphalt reduced with the increase in the R/P, R, and P concentration. In other words, adding R/P, R, and P with different dosages increased the softening point after the short-term aging happened as revealed in Figure 7. The two main reasons lead to the asphalt aging are the chemical reaction between oxygen and aromatics, and volatilization of small molecules component in asphalt. Generally, during the process of asphalt aging, resins contain polar functional group is tended to turn into asphaltenes with aggregation and condensation reaction; aromatics are tended to change through resins with oxidative polymerization reaction; saturations are comparatively steady with minor change. Components of the asphalt are usually transformed heavy directions of aromatics–resins–asphaltenes. Consequently, softening points of R/P, R, and P were raised after aging. One reason for the SI low value of R/P-modified asphalt binders with a high percentage is likely due to during the aging process, the process of swelling of rubber is still acting, that lead to accelerate the transformation of the small molecule component to asphaltenes and resin.

According to Figure 7, the softening point of neat asphalt raised by nearly 8.6 degrees after aging, whereas the addition (1% R + 5% P) increased the softening point to 7.2 degrees, suggesting that the aging resistance was enhanced. When the content of P was constant, with the increase in R content (from 1% to 7%), the increment of softening point of modified asphalt decreased obviously. When the dosage was 7% of R the softening point was less about 1.4% than that of the 1% of R content which indicating that the addition of R effectively enhanced the aging resistance of asphalt. When the concentration of R was fixed, the increase in the softening point of modified asphalt decreased with the addition of P but the law was not obvious. The results displayed that the use of R/P, R, and P had a significant impact on enhancing the aging resistance and showed lower hardening aging. Less SI values could be considered as an indicator for less change in high temperatures properties once aging [45].

#### 3.4.2. Short Term Aging Effect on the Penetration

**Figure 8:**
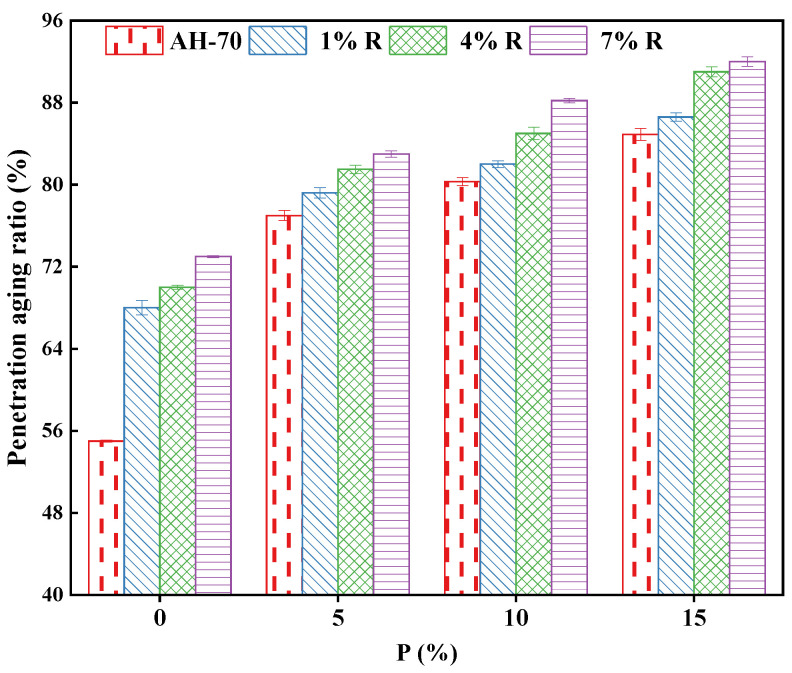
Penetration aging ratio.

The PI of asphalt reflects the change of penetration before and after aging, and its value is specified by the ratio of penetration before and after aging. As the PI increases, the better the resistance to aging is. On the contrary, the smaller the PI value, the worse the aging resistance [46]. The changes in PI of neat asphalt and R/P-, R-, and P-modified asphalt binders with various contents before and after short-term aging are displayed in Figure 8. According to Figure 8, the addition of R/P increased PI values significantly and it was positively correlated with the increase in R/P content. The addition of (7% R + 10% P) increased the PI value of neat asphalt by 60.7%, suggesting that aging resistance of asphalt was significantly improved after adding R/P modifier. With the rise of R content, the anti-aging performance enhanced gradually. However, adding different dosage of P with same content of R to asphalt binder led to increase the PI value greatly, indicating that the effect of P on the anti-aging characteristics of asphalt binder was good. Furthermore, asphalt binder modified with (7% R + 15% P) had the highest PI value, which was 36% more than that of neat asphalt. 

The enhancement of the aging resistance of R/P-modified asphalt binders is likely because of two main reasons. Firstly, the high specific surface area of R and P, leads to easy contacts with asphalt binders. Thus, an intense swelling reaction in the aging process retarded the impact of aging on the asphalt. Secondly, R, and P have internal component when it is melted through the binder. The black carbon in the P and natural contents that resist aging in R might be melted in the binder, in order to resist the oxygen and impact of thermal aging to a certain range. Thus, the act of the two could enhance the asphalt binder resistance against aging. The rise in the PI value, implying the level of aging was reduced. The neat asphalt showed the less values of PI comparing to asphalt binders modified with R/P, R, and P.

#### 3.4.3. Short Term Aging Effect on the Viscosity

**Figure 9:**
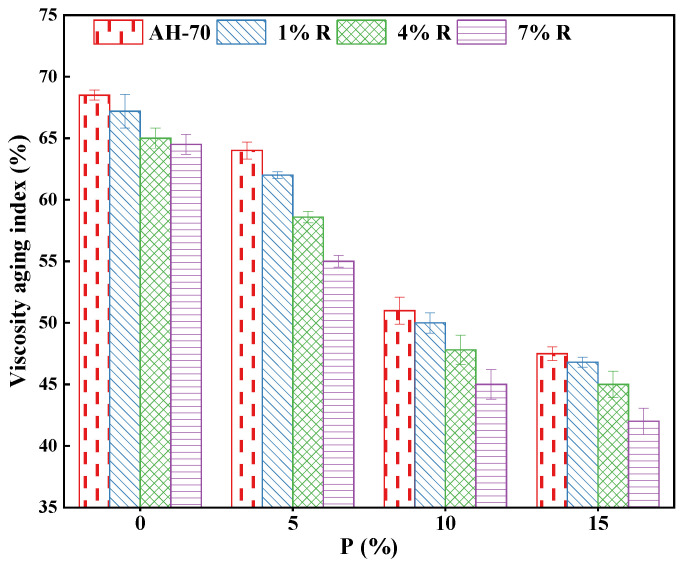
Viscosity aging index.

Figure 9 illustrates the influence of short-term aging on the viscosity of neat asphalt and R/P-, R-, and P-modified asphalt binders. In Figure 9, there are no VI values for neat asphalt and asphalt binder modified with R/P, R, and P lower than 0. This is to mention that viscosity value of neat and asphalt binder modified with R/P, R, and P were risen after the aging on short term occurred based on Equation (3). This is because of the oxidation of asphalt during the aging process, which causes an increase in phase and molecular weight. Furthermore, the addition of R/P led to significant reduction in the VI value particularly at higher dosages. When the content of P was raised from 0% to 5%, the VI is slightly decreased but it significantly changed when it reached 10%. The VI values of the neat asphalt were reduced by 7.2% and 26.1% when added 5% and 10% of P, respectively. The VI values of modified asphalt binders slightly decreased with the rise of R content from 4% to 7% as displayed in Figure 9. For 7% of R and (7% R + 10% P), the VI values of the neat asphalt were reduced by 6.5% and 34.8%, respectively. Moreover, with the rise in R/P, R, and P content, the VI value decreased which indicating that the adding of R/P, R, and P enhanced the resistance of asphalt against aging. The betterment is because naphthene aromatics were turned partially to polar aromatics and polar aromatics transformed to asphaltenes. The increases in asphaltenes resulted to increase in the viscosity. Furthermore, asphalt binder modified with (7% R + 15% P) had the lowest VI value, which was 39% less than that of neat asphalt as shown in Figure 9. The higher the content of R/P, the more obvious the improvement of the aging resistance. Asphalt binders with less VI values had the greater resistance to aging. On the contrary, the aging resistance is worse. Therefore, the asphalt binder modified with (7% R + 15% P) exhibited the best resistance against aging.

#### 3.4.4. Short-Term Aging Effect on the Rutting Factor (G*/sinδ)

**Figure 10:**
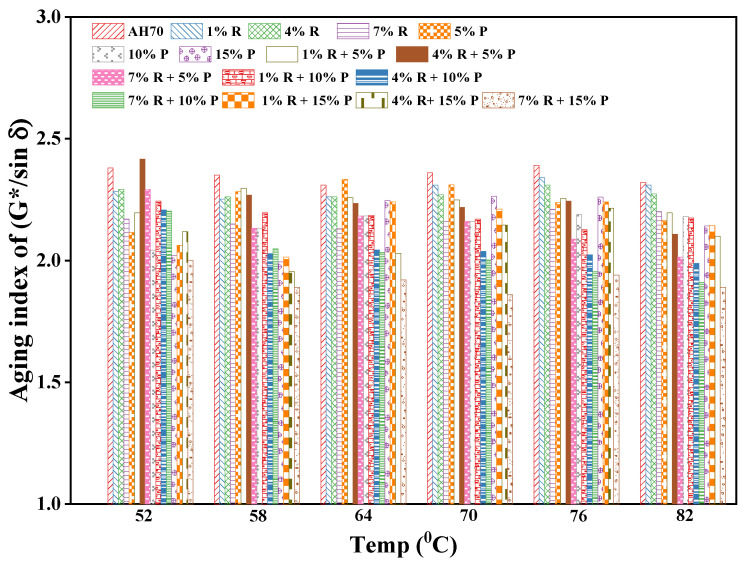
Aging index of (G*/sinδ).

Results of AIR of neat and R/P-, R-, and P-modified asphalt binders at various temperatures are demonstrated in Figure 10. The results exhibited that the addition of R/P, R, and P reduced the aging susceptibility of the binder as the AIR of R/P-, R-, and P-modified binders was lower than the neat asphalt, indicating that R/P could act as an antioxidant agent and might minimize the aging rate of binder in the field. Such behavior could be the result of the R and P particles distribution in the asphalt that was providing hindrance during the oxidation process. Moreover, the porosity and roughness of R can protect binder inside from the damage occurred by high temperature. The neat asphalt had a maximum value of AIR comparing along asphalt binder modified with R/P at different temperatures. The asphalt binder with lower AIR values displayed a low susceptibility towards aging and therefore a good resistant against aging [47]. The AIR of each binder type was more than 1 and that the process of RTFO aging led to binder hardening. Hence, an increase in R/P, R, and P content lowered the changes in G*/sin δ due to aging, and thus helped improving the resistance of the asphalt binder against aging.

### 3.5. Modified Mechanism Based on FTIR Spectroscopy

Generally, all organic components have their own infrared spectrum, and the analysis of functional groups led to the obtaining of the chemical molecular structure of asphalt. 

**Figure 11:**
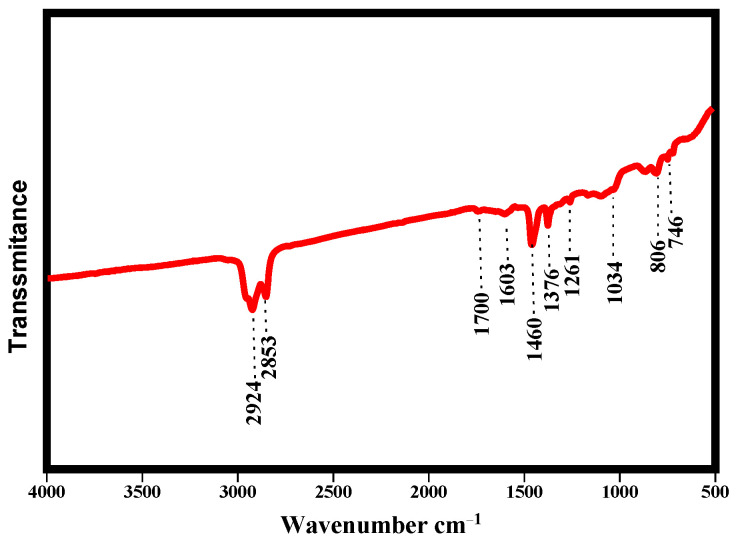
FTIR spectra of neat asphalt binder.

Figure 11 illustrates the FTIR spectra of neat asphalt. Figure 11 shows that absorption band at the functional group region were occurred at 2924 cm^−1^, 2853 cm^−1^, 1700 cm^−1^, 1603 cm^−1^, 1460 cm^−1^, and 1376 cm^−1^. Based on the absorption peaks, there are two regions which are the functional group region and the fingerprint region. The absorption peaks within 4000 cm^−1^~1300 cm^−1^ are corresponding to the functional group region, whereas excepting for a single bond vibration, the absorption peaks within 1300 cm^−1^~600 cm^−1^ are corresponding to the fingerprint region. For the neat asphalt, Figure 11 displays that the FTIR spectra of neat asphalt had main peaks at 2924 cm^−1^ and 2853 cm^−1^ due to the symmetric stretching of -CH_2_ and -CH_3_ groups, besides the symmetric -C-H stretching of -CH_2_ group of hydrocarbon chain. The presence of the absorption band at 1603 cm^−1^ is associated with -C=C- vibration in aromatics. The appearance of the absorption band at 1460 cm^−1^ corresponding to -C-H- asymmetric deformation of -CH_3_- and -CH_2_-, and that appearance at 1261 cm^−1^ and 1376 cm^−1^ is because of frame vibration of -(CH_3_)_3_C-R- group and -CH- symmetric deformation vibration in -CH_3_-. As the asphalt is formed of hydrocarbons with 10% of hydrogen and 81% of carbon, the occurrence of all these peaks was anticipated. The presence of peak at 1034 cm^−^^1^ corresponds to S = O vibration peak and is commonly applied to characterize the evolutions of the chemical structure of aged asphalt binder. Moreover, the small peaks in region between 671 cm^−1^ and 871 cm^−1^ are typical C–H vibrations of the benzene ring [48]. Obtained results from the FTIR spectra of neat asphalt confirm that asphalt mostly contained aromatics, cycloalkane, and alkanes.

**Figure 12:**
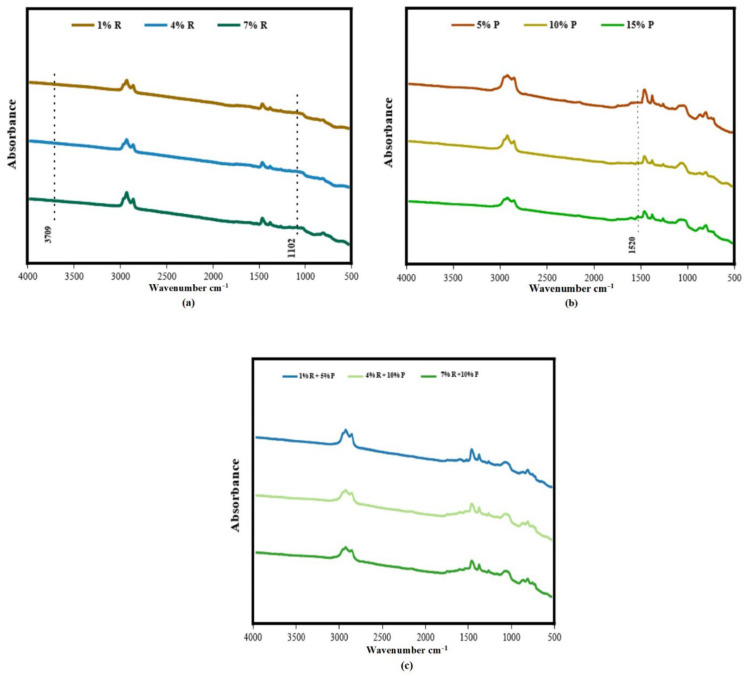
FTIR spectra of (**a**) R-modified asphalt, (**b**) P-modified asphalt, (**c**) (1% R + 5% P), (4% R + 10% P), and (7% R + 10% P)-modified asphalt.

The FTIR spectra of R-modified asphalt, P-modified asphalt, and (R/P)-modified asphalt were measured, respectively, as revealed in Figure 12. In the case of R-modified asphalt, Figure 12a exhibits that, the great impacts of adding different dosage of R to the asphalt binder are the appearance of associated with hydroxyl functional group about 3709 cm^−1^, that indicate there is a reaction between silica and asphalt binder when adding of R. As a previous study [49] prove that the absorption bonds of O–H were come from silanol in Si(OH)_4_ outputted from the H–OH vibration. Si(OH)_4_ presence is revealed at 1102 cm^−1^, implying the bond of Si–O–Si form via deformation of the vibration of Si-O, which previous study proved that [50]. The appearance of SiO vibration could be detected in result, displaying good non-bonded interaction of the SiO tetrahedra with asphalt binder. That is to say that the SiO vibration suggest that intensive interaction between the R and binder, and distortions in the SiO tetrahedra.

For the P-modified asphalt, Figure 12b shows that, a new peak was appeared at 1520 cm^−1^. As Liu H. [51] et al. mentioned, this peak is due to frame vibration of benzene ring occurred because the adding of P to asphalt binder. Based on this, the modification mechanism of P was mainly physical modification with no chemical reaction. These results are same line with the findings of previous study was conducted by Zhang et al. [52]. They stated that asphalt binder modified with polymers were totally physical modification without a chemical reaction. Moreover, Liu H. et al. [51] studied the impact of P on asphalt binder and concluded that physical interaction as main modification mechanism of the asphalt binder. Abdelrahman et al. [53] proposed that one of the main modification mechanisms through using P is the swelling of P particle by asphalt binder fraction. For the R/P-modified asphalt, the FTIR spectra of (1% R + 5% P), (4% R + 10% P), and (7% R + 10% P) are revealed in Figure 12c. Figure 12c displays that R/P composites had all the peaks of neat asphalt, R-modified asphalt, and P-modified asphalt. It is important to mention that no additional peak presence was observed. 

### 3.6. Aging Mechanism of R, P, and R/P

**Figure 13:**
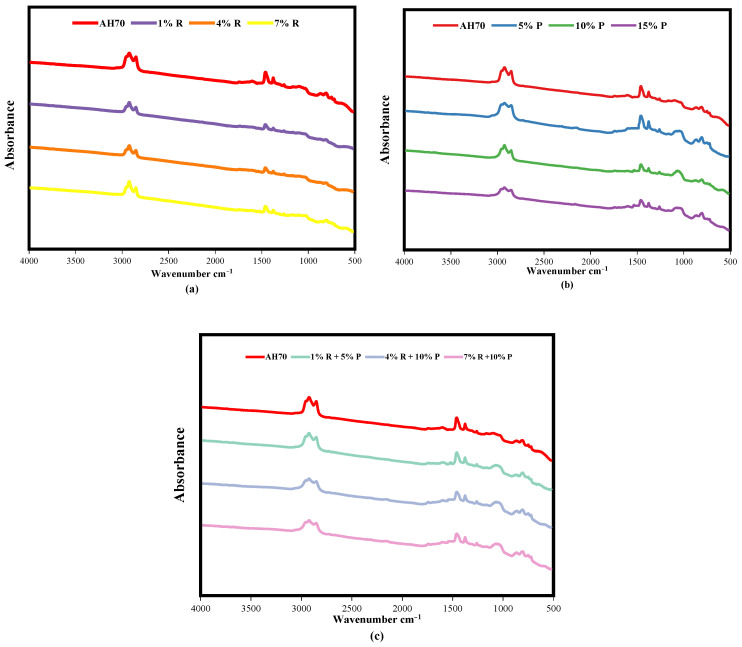
FTIR spectra of (**a**) R-modified asphalt, (**b**) P-modified asphalt, (**c**) (1% R + 5% P), (4% R + 10% P), and (7% R + 10% P)-modified asphalt after RTFO aging.

The FTIR spectra of neat asphalt and modified asphalt after aging took place are revealed in Figure 13.

From Figure 13, it is clear to observe that, no new characteristic peaks and functional groups presence on (neat asphalt, R-, P-, and R/P-modified asphalt binders) after RTFO aging took place, only the peak intensity varied. This indicates that during the short-term aging process, there are no change in the functional groups of the asphalt binder. The absorbance spectra were selected, and baseline correction was used for FTIR spectra to study the aging mechanism of R, P, and R/P through determine the difference between peak values before and after aging, that represent the aging degree [54]. The higher the peak value was, the more advanced the aging of R, P, and R/P was. It is important to mention that (OMNIC) program and (Spectragryph v1.2.15) software [55] were used to determine peak height and area.

Table 4 demonstrates tendency of varies changes in the value of peak of functional group at different dosages of R, P, and R/P (1% R, 7% R, 5% P, 10% P, (1% R + 5% P), and (7% R + 10% P)).

Table 4 shows that the intensity of absorption peak for various functional groups varied with the different content of R, P, and R/P. Moreover, after aging took place the peaks of light components reduced, which suggested that the light components in the asphalt during the aging are volatilized. Asphalt binder modified with (7% R + 10% P) had the lowest peak difference followed by asphalt binder modified with 10% P, (1% R + 5% P), 5% P, 7% R, and 1% R, respectively. As mentioned earlier, the higher the value of the peak was, the more advanced the aging of R, P, and R/P was. Table 4 suggests that during the process of aging, R, P, and R/P may well prevent volatilization of light components. The porous structure of the R and the high surface area of R and P could raise the interfacial force between R and P particle and asphalt, and it inhibit volatilization of light components. 

To quantitatively analyze the functional group evolution of asphalt binders after RTFO aging took place, the carbonyl index (*I_C=O_*) and sulfoxide index (*I_S=O_*) were determined according to Equations (5) and (6), respectively [56,57,58]. Moreover, the $I_{C=O}$ difference before and after aging was determined from Equation (7). The higher $\;I_{C=O}$ difference (Δ$I_{C=O}$) between the aged and un-aged sample, the deeper the aging of the asphalt binder is [35].

(5) $$I_{C=O}=\frac{A_{1700}}{\sum A}$$

(6) $$I_{S=O}=\frac{A_{1034}}{\sum A}$$

$$\sum A=A_{2924+2853}+A_{1700}+A_{1603}+A_{1460}+A_{1376}+A_{1034}+A_{806}+A_{746}$$

*A* represents the area of each spectral band.

(7) $$\Delta I_{C=O}=I_{c=O_{aged\;sample}}-I_{c=O_{unaged\;sample}}$$

The structural indices of asphalt binders before and after aging took place are tabulated in Table 5. As shown in Table 5, the carbonyl and sulfoxide steadily increased for all asphalt binders at RTFO aging. In addition, it is clear to observe that with the addition of R, P, and R/P the carbonyl and sulfoxide decreased, which suggested decreased asphalt oxidation. The increase in carbonyl and sulfoxide group functions after aging indicated that the asphalt was oxidized and thus the oxygenated structures were formed during the RTFO aging. Furthermore, the $I_{C=O}$ value of neat asphalt increased by 0.006070 after aging, which was the highest increment of $I_{C=O}$ compared to all modified asphalt binders. In addition, the neat asphalt displayed a higher increment of I_S=O_ value compared to R, P, and R/P-modified asphalt binders. The degree of aging is not the same at different concentrations of R, P, and R/P, but still exhibited the highest aging resistance due to minimum structural indices. The enhancement of aging resistance of R-, P-, and R/P-modified asphalt binder compared with neat asphalt could be due to the following mechanisms: the porous structure of the R which could adsorb light components in asphalt. Additionally, P particles absorbed the light components in the asphalt that lead to very few reactive asphalt molecules could oxidized. Moreover, during the aging process, the light fractions of asphalt that absorbed by P particles may be released through the asphalt to compensate the asphalt molecules that could be lost at aging stage. In addition, polymer chains from the rubber network could play an important role as retardant to inhibit the penetration of oxygen molecules into the asphalt. Therefore, polymer chains were preferably attacked via oxygen, which led to delay the reaction between oxygen with polar bitumen molecules. 

In accordance with Table 5, the obtained Δ$I_{C=O}$ values after RTFO aging were 0.006070, 0.002950, 0.002608, 0.002155, 0.001133, 0.001491, and 0.000866 for neat asphalt, asphalt binder modified with 1% R, 7% R, 5% P, 10% P, (1% R + 5% P), and (7% R + 10% P), respectively. It appears that with the aging degree deterioration, Δ$I_{C=O}$ was regularly magnified for all asphalt binders, the obtained results are corresponded with trend of aging indices. Moreover, the neat asphalt had the highest Δ$I_{C=O}$ value after RTFO aging compared to R-, P-, and R/P-modified asphalt binders, whereas asphalt binder modified with (7% R + 10% P) had the lowest Δ$I_{C=O}$ value, further indicating the superior anti-aging characteristics of R-, P-, and R/P-modified asphalt binders. In summary, the R, P, and R/P material are good modifiers in preventing oxidization reaction in the asphalt binder.

### 3.7. Morphology

**Figure 14:**
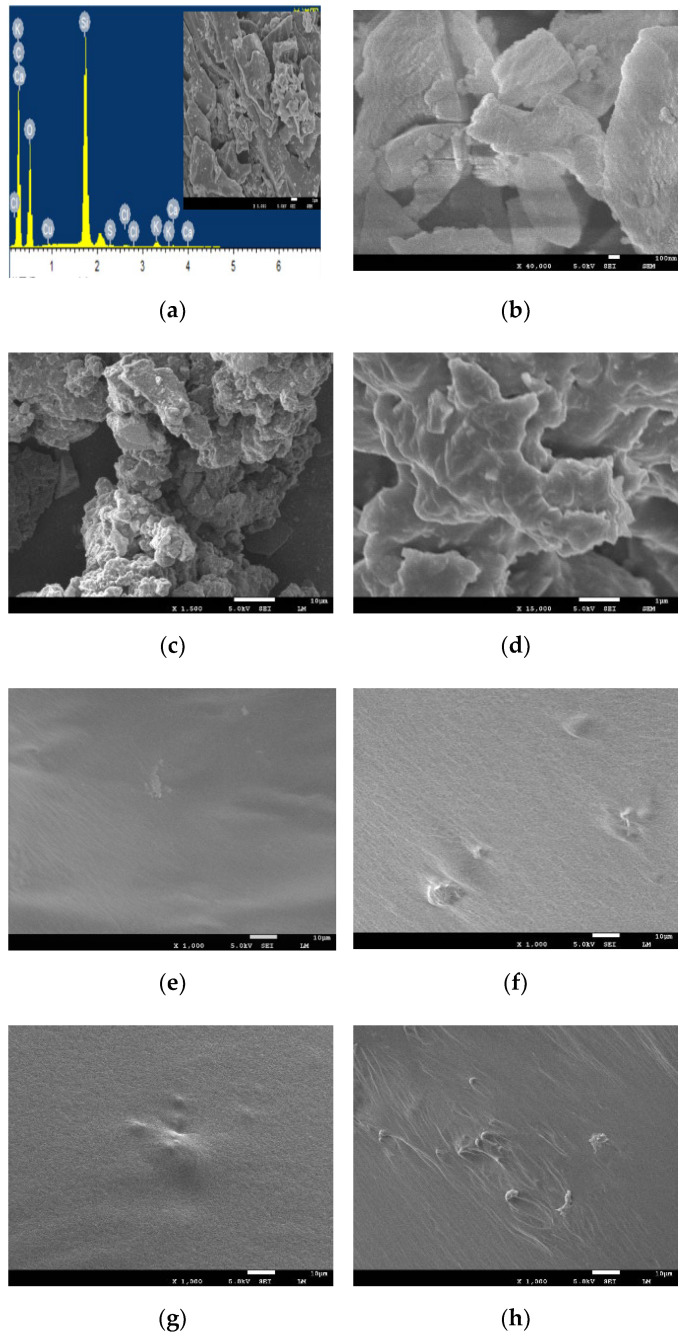

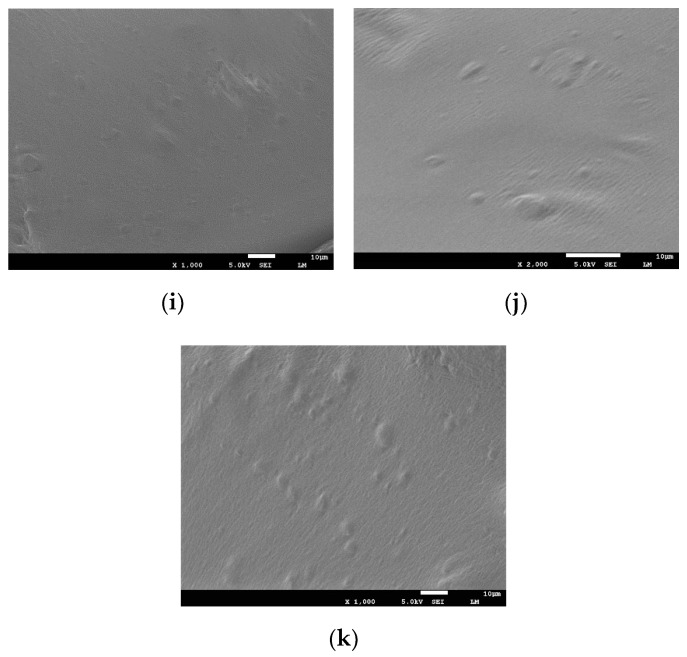
SEM graph of (**a**) R with EDS analysis, (**b**) R, (**c**,**d**) P, (**e**) 1% R-modified asphalt, (**f**) 7% R-modified asphalt, (**g**) 5% P-modified asphalt, (**h**) 15% P-modified asphalt, (**i**) (1% R + 5% P)-modified asphalt, (**j**) (7% R + 10% P)-modified asphalt, and (**k**) (7% R + 15% P)-modified asphalt.

For more measure to the micro-structure of asphalt modified with R, P, and R/P, the scanning electron microscopic (SEM) was used to assess the morphology of the modified asphalt binder. Images obtained by using the SEM are revealed in Figure 14 for R, P, different dosages of R-modified asphalt, P-modified asphalt, and R/P-modified asphalt, respectively.

From Figure 14a,b, it can be seen that coarser R particles of irregular shape with very porous structure and various layer system (exterior layer, interior layer, and interfacial layer) that could be identified from the cross-section SEM image of R particles. This result in good agreement with pervious study [36] reported that interfacial layer consists of a crisscross mesh of chips and abundant pores. Inspection of Figure 14a,b indicates both of interior layer and exterior layer had a rough surface. In addition, the micro R particles surface consists of micro sheets and microchips in three-dimensional reticular distribution as displayed in Figure 14a,b. These micro sheets and microchips contain white columnar SiO_2_ crystal whiskers on their surface and edges. From Figure 14c,d it can be seen that P has an irregular shape with a high specific surface, which could raise the contact area between P particles and binder and improve the swelling impact of P in asphalt [59]. Thus, the P could absorb additional light components in binder during the swelling process.

Figure 14e,f show that R is well dispersed in asphalt binder and this phenomenon can be explained due to the white columnar SiO_2_ crystal whiskers on the edges and surface of the micro sheets and microchips could create attraction between asphalt binder and SiO_2_ crystal whiskers. In other words, the activated amorphous SiO_2_ that in R can strongly react with bitumen and produce an efficient filling structure, that lead R particles to disperse equably in the composite of modified asphalt. It is important to mention that the interior layer, exterior layer, and interfacial layer structure and micro-pores in R have positive effects on the adhesion characteristics of modified asphalt. Regarding the R pozzolanic characteristics, it is anticipated to make a better bond with the bipolar particles of asphalt.

Figure 14g,h show the SEM images of P-modified asphalt, as the accumulation of P particles in the asphalt binder can be seen in Figure 14, which suggests a good homogeneity and dispersion of the modified asphalt binder [60,61]. The surface roughness of P-modified asphalt raises because of the gathering of P particles and led to good bonding within the asphalt binder that led to improvement in the physical characteristics of the P-modified asphalt binder. SEM images of (R/P)-modified asphalt from Figure 14i–k indicate the presence of P and R particulates, which FTIR also confirms. SEM confirms the compatibility among P, R, and asphalt binder in the producing of appropriate composite. This interfacial adhesion and compatibility among P, R, and the asphalt binder are responsible for improving the hot mix asphalt performance through boosting the binding characteristics of the asphalt binder.

## 4. Conclusions

Based on the test results of this research, the following conclusions can be drawn.

1. Asphalt binder after mixing with R and P revealed an increase in softening point, and viscosity, whereas it showed a decrease in the penetration, indicating the asphalt binders become stiffer and have a good ability to resist permanent deformation.
2. The G*/sin (δ) values obtained from DSR measurement were directly proportioned to the modifier concentration. Therefore, resistance to permanent deformation and elastic response of the binder were enhanced by increasing the G*, and reducing the (δ).
3. The addition of R/P caused the increase in the (PI), whereas the (SI) and (VI) decreased which suggest the asphalt binders modified with R/P had good performances of aging resistance comparing to the neat asphalt. Furthermore, the decrease in the (AIR) of asphalt binder modified with R/P compared with the neat asphalt suggest that R/P had good influences on the aging resistance of the asphalt binders.
4. Based on FTIR spectroscopy test, the obtained results showed that R/P could enhance the aging resistance of asphalt binder. Compared with neat asphalt, modified asphalt binders with different dosages of R/P exhibited better aging resistance. Therefore, the R/P-modified asphalt is suitable for using in pavement construction in terms of preventing the reduction in service life occurred by aging.
5. The new hybrid modified asphalt (R/P) with (7% R + 10% P), considered as the optimal choice for excellent overall performance of modified asphalt.
6. The apparent morphologies revealed that the R particles have a loose and porous structure, that lead to bring it good adsorption to the asphalt binder, whereas the P particles have a high specific surface and irregular shape. Based on SEM images, a homogeneous mix was formed due to the uniformly dispersed of R, P, and R/P within the asphalt binder.

The authors will study the impact of long-term aging on the asphalt binder modified with R and P in the forthcoming study, as well as the effect of R and P as modifiers on the low- and intermediate temperature performance of asphalt binder.

## Figures and Tables

**Table 1:** Properties of asphalt binders.

Test	Result
Flash point (°C)	326
Softening point (°C)	47
Ductility (cm)	>150
Penetration depth (0.1 mm)	72
Viscosity at 135 °C (Pa s)	1.03
Specific gravity (g/cm^3^)	1.04

**Table 2:** Physical characteristics and Chemical’s constitution of rice husk ash.

Physical Properties	Specific Gravity (g/cm^2^)	2.34
	Silicon dioxide SiO_2_ (%)	86.84
	Potassium oxide K_2_O (%)	3.76
Chemical’s constitution	Calcium oxide CaO (%)	1.60
	Aluminum oxide Al_2_O_3_ (%)	0.61
	others (%)	7.19

**Table 3:** Crumb rubber powder properties.

	Break Strength (MPa)	13.2
Physical Properties	Moisture content (%)	0.61
	Density ratio (g cm^−3^)	1.13
	Acetone Extract (%)	2
	Carbon black content mass (%)	34.1
Chemical’s constitution	Rubber hydrocarbon content mass (%)	59.8
	Ash content (%)	4.1

**Table 4:** Peak values for the featured functional groups of modified asphalt binders.

Wave Number (cm^−1^)		2924	2853	1603	1376
1% R	Before aging	0.447665	0.375135	0.056410	0.145101
	After aging	0.341323	0.298921	0.040428	0.064079
	Difference	0.106342	0.076214	0.015982	0.081022
7% R	Before aging	0.386602	0.239829	0.028799	0.099733
	After aging	0.327356	0.190112	0.019795	0.054167
	Difference	0.059246	0.049717	0.009004	0.045566
5% P	Before aging	0.341323	0.238921	0.027499	0.064079
	After aging	0.288448	0.189702	0.019520	0.048269
	Difference	0.052875	0.049219	0.007979	0.015810
10% P	Before aging	0.313854	0.225870	0.027180	0.057306
	After aging	0.26349	0.179880	0.019377	0.043490
	Difference	0.050364	0.045990	0.007803	0.013816
1% R + 5% P	Before aging	0.330625	0.237292	0.027326	0.062932
	After aging	0.278998	0.188452	0.019492	0.047371
	Difference	0.051627	0.04884	0.007834	0.015561
7% R + 10% P	Before aging	0.277088	0.161740	0.020933	0.041933
	After aging	0.228769	0.129258	0.013900	0.039239
	Difference	0.048319	0.032482	0.007033	0.002694

**Table 5:** Group indices of various modified asphalt binders before and after aging took place.

	Chemical Bonds				
	$I_{C=O}$		$I_{S=O}$		$\Delta I_{C=O}$
	Before Aging	After Aging	Before Aging	After Aging	$Aged\;I_{c=o}-Unaged\;I_{c=o}$
neat asphalt	0.004215	0.010285	0.007024	0.011861	0.006070
1% R	0.004322	0.007272	0.009165	0.011251	0.002950
7% R	0.004279	0.006887	0.009228	0.010842	0.002608
5% P	0.003856	0.006011	0.009132	0.010211	0.002155
10% P	0.003898	0.005031	0.008978	0.009915	0.001133
1% R + 5% P	0.003841	0.005332	0.009047	0.009989	0.001491
7% R + 10% P	0.004028	0.004894	0.008863	0.009066	0.000866

## Data Availability

Not applicable.
